# Complementary Approaches to Existing Target Based Drug Discovery for Identifying Novel Drug Targets

**DOI:** 10.3390/biomedicines4040027

**Published:** 2016-11-21

**Authors:** Suhas Vasaikar, Pooja Bhatia, Partap G. Bhatia, Koon Chu Yaiw

**Affiliations:** 1 Integrative Biology, Baylor College of Medicine, Houston, TX 77030, USA; 2 School of Biological Sciences, Indian Institute of Technology, Delhi 110016, India; poojabhatia.iitd@gmail.com; 3 Department of Pharmaceutics and Pharmaceutical Microbiology, Usmanu Danfodiyo University, Sokoto 840231, Nigeria; partapgbhatia@gmail.com; 4 Experimental Cardiovascular Research Unit, Department of Medicine-Solna, Center for Molecular Medicine, Karolinska Institute, Stockholm 17177, Sweden; koon.yaiw@ki.se

**Keywords:** drug discovery, drug design, drug targets, repositioning, molecular imaging

## Abstract

In the past decade, it was observed that the relationship between the emerging New Molecular Entities and the quantum of R&D investment has not been favorable. There might be numerous reasons but few studies stress the introduction of target based drug discovery approach as one of the factors. Although a number of drugs have been developed with an emphasis on a single protein target, yet identification of valid target is complex. The approach focuses on an in vitro single target, which overlooks the complexity of cell and makes process of validation drug targets uncertain. Thus, it is imperative to search for alternatives rather than looking at success stories of target-based drug discovery. It would be beneficial if the drugs were developed to target multiple components. New approaches like reverse engineering and translational research need to take into account both system and target-based approach. This review evaluates the strengths and limitations of known drug discovery approaches and proposes alternative approaches for increasing efficiency against treatment.

## 1. Introduction

**Figure 1:**
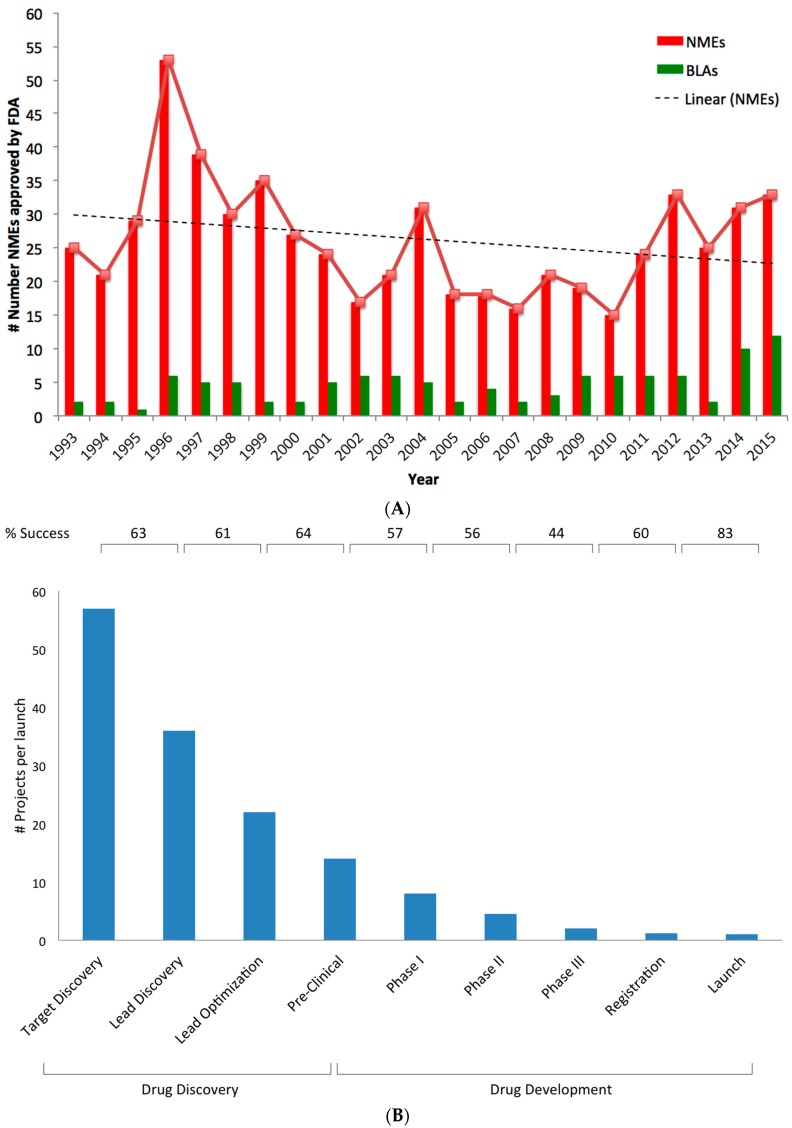
(**A**) U.S. Food and Drug Administration (USFDA) approved new molecular entities (NMEs). Number NMEs approved by the USFDA; (**B**) Attrition rate across the different stages of drug discovery and development phase. Around 80% of attrition rate was observed in drug discovery phase. Whereas more than 90% attrition was observed in drug development phase. In summary, ~1 in 50 projects reach market from discovery phase [4].

During the past decade, an increasing number of innovations based on Genomics, Proteomics, Bioinformatics, Pharmacology, and Genetics have led to rise in innovative drugs. However, despite impressive advances in technologies there has been a decline in new molecular entities [1,2]. In last few decades, a low number of submissions for NMEs (new molecular entities) by the pharmaceutical and biotechnology industry was observed [3]. NMEs are often innovative new products that serve previously unmet medical needs or otherwise significantly help to advance patient care and public health (www.fda.gov). Figure 1A depicts the number of NMEs filed for approval and approved by the U.S. Food and Drug Administration (FDA). In the year 1996, the US FDA approved 59 NMEs, but after that till 2002 a steady decrease in approved drugs has been observed. In the year 2004 and 2012 the US FDA approved 36 and 39 NMEs respectively. However, in last decade the number remained lower than approved in the year 1993 (27 NMEs) except 2011 (30 NMEs). In the year 2015, 45 new drugs were approved which was a higher number compared to last decade approvals.

The reasons for this decline were uncertain, but it was suspected that drug discovery approaches and development remains major factor [2,5,6]. Kola and Landis (2004) [5] have observed that the attrition rate depends on the stage of development and therapy area. For example, adverse pharmacokinetic and bioavailability results were said to be major cause of attrition in 1991, whereas these factors attributed to less than 10% attrition in 2000. The attrition rate across the different stages of drug Discovery and Development phase was shown in Figure 1B. The failure rate for the drugs entering from Phase II to Phase III increases up to 50% [7,8]. Thus, overall, it was observed that only a drug out of 10 that FDA approved entered clinical tests. Though, the proliferation in number of NMEs relies on the submission of number of applications, the drug development costs (including cost of failure) have risen to an estimated average of $1.8 billion per approved drug in 2013 versus $800 million in 2003 [9]. It was observed that the commercial value of novel targets declined in last decade. However, for novel targets with extensive in vivo validation studies have been successful. This suggests that target validation is an important step for novel target to reach preclinical development. Haak (2004) has reported that the estimated probability for a novel target is only 3% compared to 17% for a known target to reach preclinical stage [10]. Since the 1990s, the predominant approach used to identify novel targets was target-based drug discovery, which lured the pharma sector. The analysis by Swinney et al. (2011) shows that between 1999 and 2008 about ~100 NMEs among 259 agents were discovered using target-based approaches [11]. The target-based approach defines the target and associated specific molecular mechanism or mode-of action (MoA) to be targeted by the treatment. Despite the fact that target based approach delivered a number of new drugs, it has not resulted in high success rate [11,12].

From a commercial perspective, the low enrichment of drug targets at preclinical stage from high input resource represents risky business strategy. If we consider that every drug discovery takes minimum 2 years then for N number of drugs it would take 2N years for evaluation. In short for 25 novel drugs at least 50 years of research needs to be carried out. Thus, identifying the novel invalidated targets by pharmaceutical industry seems expensive proposition. Whereas, focusing on known targets and using existing drugs in new indications have limitations. For last few years, research was mainly focused on target-based approach limitations [13,14,15]. However, the question is whether the given approach more suitable or there is need of new approaches for the identification of novel treatments. The purpose of this review is to investigate existing and new approaches towards drug discovery and improve pharma productivity.

### 1.1. Target-Based Drug Design

**Figure 2:**
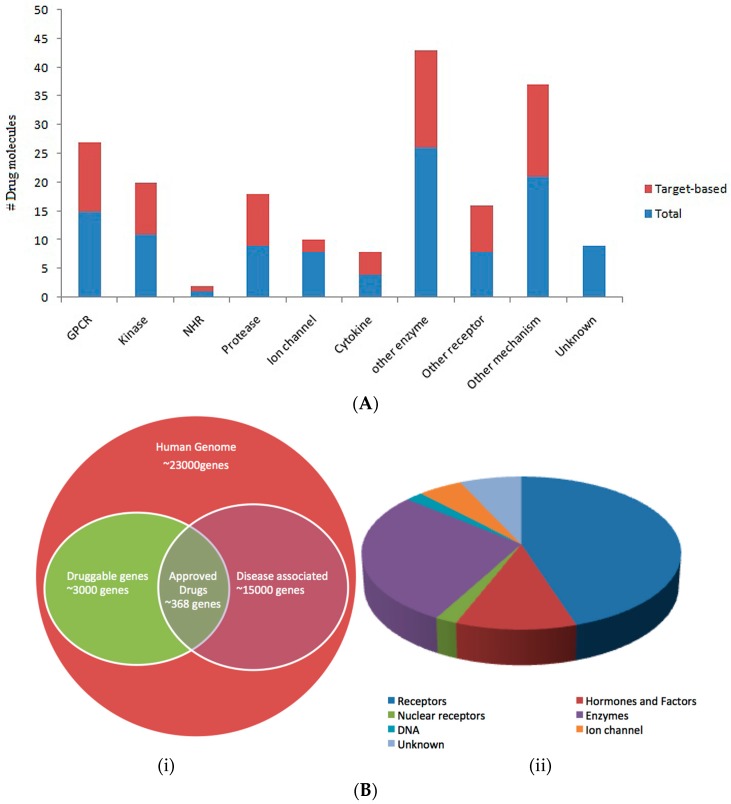
(**A**) Classification of drug targets based on FDA approved drug molecules [16]; (**B**) Druggable genome. (i) The Venn diagram shows the subset of human genome associated with disease and available drugs targeting subset of genes. (ii) Classification of the drug targets encoded by the human genome.

A target is usually a single gene, gene product or molecular mechanism towards disease prognosis. According to Sams-Dodd et al. (2006) the targets can be divided into two classes: genetic or mechanistic target [16]. The genetic target represents the genes or gene-derived products, whereas the mechanistic targets represents receptors or enzymes. The general classification of drug targets was shown in Figure 2A. The target based drugs often modulate the function of the proteins, or total inhibition in case of pathogens. Antimicrobial drugs are mostly target based and the targets should (i) be pathogen specific; (ii) essential and having unique function; (iii) can be inhibited. While selecting from the available receptor structures another point to be kept in mind is that it should have (i) maximum number of alpha helical structures, this provides rigidity to the structure; (ii) less number of beta-sheets, as this renders active site flexible; (iii) less number of loops. It differs from physiological based approach in that it often develops disease-mimicking properties in given model and demonstrates appropriate drugs are effective in the disease model. This drug discovery paradigm has delivered effective drugs against many diseases including psychosis, depression. However, the problem lies in identifying the Mode of Action of effective drugs or compounds [16,17].

The genetic target focus on the genomic content of the pathogen or cell, modulation of which alters the genetic program [18,19]. It should fulfill the two conditions; first the diseases caused by genetic mutations or increased disease risk attributed to a genetic factor. Second, it must contribute to disease prognosis. Use of genetic target for drug discovery has been limited as most of the diseases are multifactorial. Thus, the nucleic acids can be appropriate target [20], but because of its complexity, nucleic acid targeted drug design has not been major path opted in pharmaceutical discovery programs [21]. Among genetic material, RNA has low structural complexity and high flexibility. RNA was shown to be a more effective target because of its functions, low resistance, highly conserved functional domains, and unique RNA binding motifs among closely related molecules [18,22]. The well-known examples of RNA-targeting antibiotics are aminoglycosides and macrolides that inhibit prokaryotic translation. Further, comparative analysis of genome sequence of bacterial pathogens available on public database enables drug discovery approach [23,24]. The approach relies on small genome size of parasite code for few numbers of proteins. For example, enteric pathogen *Escherichia coli* O157:H7 codes for Shiga toxins 1 and 2 (Stx1 and Stx2), which cause hemorrhagic colitis and hemolytic-uremic syndrome [25].

Mechanistic target represent biomolecule, inhibition of which was sufficient to obtain significant therapeutic effect. The drug targets were identified based on biological observations and clinical findings. For example, receptor or enzyme altered in disease condition. It also includes the membrane-associated proteins, kinases identified in disease models. Membrane proteins such as receptors and ion channels are key molecules in the membrane that account for up to two thirds of known druggable targets, thus, represent an important domain for pharma. For example, GPCR (G protein-coupled receptors) account for over 50% of all human drug targets in cancer, cardiovascular, metabolic, CNS and inflammatory diseases. Another class of proteins includes kinases, which represent largest enzyme family encoded by human genome [26]. Though kinases share structural similarity, which limits the development of selective drug. However, the development of SB203580 and SB202190, both p38 antagonists, by SmithKline Beecham in the early 1990s demonstrated that the identification of kinase inhibitors capable of selectively modulating therapeutically relevant, biological signaling pathways was possible [27].

### 1.2. Target Validation

The volume of genomic information delivered a few potential novel drug targets, which include G-protein–coupled receptors (GPCRs) and transport proteins. However, most of potential targets remained unexplored for their therapeutic application due to complex validation process. Target validation can be address by gene knockouts, dominant negative mutants, antisense technology, expression profiling or proteomics [28]. These methods can give limited information, depending upon the animal models selected for the human disease. Among animal models, Zebrafish is easier to bred and maintain thus, can be part of higher-throughput analysis [29,30]. However, there were limitations on its use for human diseases for example psychiatric diseases. Other than Zebrafish, mice have also been used widely as a model for human diseases. Mouse knockouts have generated more useful information than any other models [31].

According to single gene disorder theory, single gene linked to disease prognosis [32]. However, its very difficult to prove that its causative role in particular disease progression. In the technique in vivo gene knockout, gene(s) are deleted or disrupted to stop their expression. This method assumes knocking down a particular gene was same as inhibiting its expression by specific inhibitor, thereby, could provide a good quality understanding of gene in vivo. Thus, effective use of gene knockout technology in disease models can focus on drug targets and any side effects before time. However, single disorders are not common. A real challenge lies in linking the causative gene with human disease. A given gene can have multiple roles or multiple genes can have a single role [33]. Human genome contains ~25 thousand genes and can express 15–50 thousand proteins in different tissues in differential amount. Hence, most of drug molecules pertaining to complex diseases fail to enter clinical trials. Another common approach to target validation was to examine altered gene expression level in a diseased tissue in comparison to normal tissue. Identifying this altered expression level was the crucial step to discover the novel potential drug targets. The assumption here was that the altered gene expression was a part of the disease process [34]. The possibility might be altered expression was as a result of disease. Not only the expression pattern but small interfering RNA (siRNA) techniques, changes in regulation, interacting partners may provide suitable approach to identify targets than traditional drug discovery approach [35].

### 1.3. Druggability

The concept of druggability refers to potential of protein or biomolecule to be modulated by drug-like compounds [36]. The ideal drug should possess high potency, low dosage, high sensitivity, few adverse effects and low production cost. However, the challenge was to identify drugs with such properties. In human, among ~25,000 genes only few are disease relevance genes which have been estimated to be between 3000 and 10,000 [37]. However, it was not the true number, as drugs may have higher number of binding site than number of druggable drug targets. This can be explained as single gene can result in different transcripts via alternative splicing, post modifications lead to expanding druggability. Figure 2B depicts druggable pools of targets.

Currently market addresses small number of drug targets. Hence pharma companies have lot of available pool to exploit. It was observed that industry evaluates only around three new druggable targets each year [38]. This rate may take industry another >150 years to launch drugs against available targets. Industry now has to change approach of “one drug, one target” and start to look at multi target drugs like Aspirin.

## 2. Challenges in Pharma and Possible Solutions

To bring new drug molecule in the market, the drug industry spends considerable amount of investment (average 16%) in R&D [3,38]. Among top 20 R&D spenders in the world, 8 pharma companies have been found to be in the Global Innovation Index list (www.globalinnovationindex.org). For example, Eli Lilly spent 23% of its revenues on R&D, Pfizer 13.5%, Roche over 19%, Merck over 17%, and AstraZeneca over 16%. Although the amount spent by drug makers has increased over a decade but the number of drug molecules that entered clinical trials failed to come in market (www.nsf.gov). In 2004, FDA released a report “Innovation or Stagnation?—Challenge and Opportunity on the Critical Path to New Medical Products” [1] to address the productivity deficit in new drug development (www.fda.gov). This report suggests need of product development toolkit that includes both computer based predictive models and animal models to improve predictability and efficiency of new drug.

Finding an optimal approach to drug development is a difficult task. Despite an expanding universe of drug targets, it is difficult to determine which proteins are involved in a disease [33,39]. If one succeeds in the identifying drug target, there is no assurance of the drug molecule binding to given the protein. Hence, 95% drugs fail to enter clinical trials contributing to high drug costs. It was observed that over 90% of drug development cost is incurred in Phase III thus, making it the most expensive stage [40]. Because of the enormous cost and risk involved in Phase III trials, pharma industry try to avoid research on chronic conditions or rare diseases. In addition, the present regulatory process in Phase III trials force drug industries to take enormous financial risk and unnecessary delay, preventing small start-ups biotech companies from challenging dominant pharma industries. Thereby, causing threat in terms of drug development, patient’s illness and cost on healthcare.

It has been estimated that ~200 drugs get stalled annually in clinical development (www.ddw-online.com). This may be attributed to limitation of target-based approach in proper target validation. In early drug discovery program many companies assume that target is well enough identified and validated. However, at the end of optimization the additional proof-of-principle studies only take around 3–5 years. Thus, there lies an inherent risk in the process, as many factors and efficacy in different disease models may change over the years. This means underestimation of the complexity of target, leading to the failure of drug in clinical trials, loss of number of years of research, money and resources. Another factor contributing to reduction in productivity was druggable targets. Many industries pursue similar target at the expense of good druggable target. How can we find druggable target for a particular disease and drug compound binding to it from universe of structures. These structures eventually should disrupt the particular protein-protein interaction or inhibit its biological activity. Combinatorial chemistry and high throughput screening (HTS) techniques have given partial solution for screening, however, these provides many hits but very few lead [41]. New approaches like reverse engineering, high-throughput screening paradigm were being focused to identify the leads [42,43]. One way of developing innovative medicines was by targeting unknown druggable targets. Currently industries are stepping into synthesis of small molecules that can target fraction of genome coding for protein.

## 3. Complementary Approaches

### 3.1. Multi-Target Drugs

**Figure 3:**
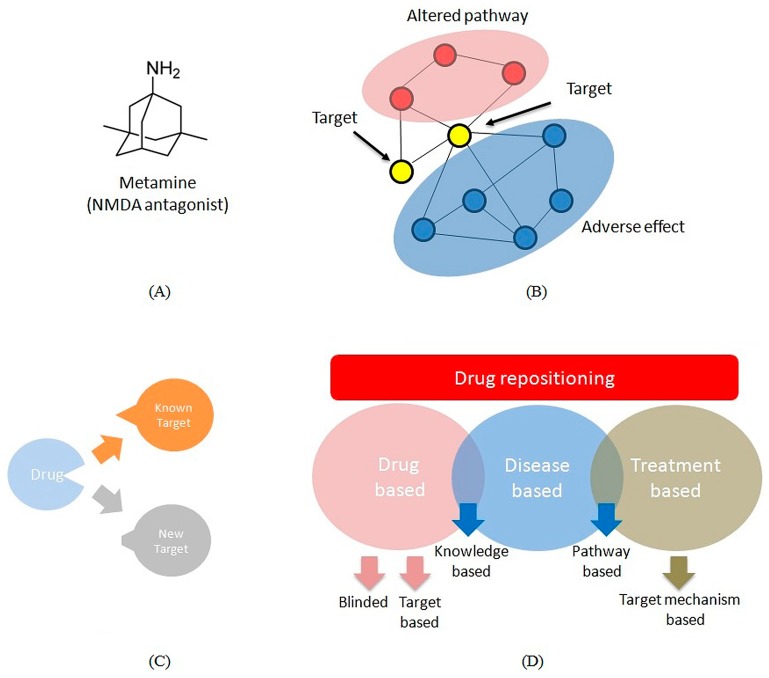
Multi-target drugs. (**A**) The chemical structure of metamine, only multi-drug molecule used to treat Alzheimer’s disease patients; (**B**) Proposed mode of action of multi-target drugs through weak-linkage of networks. Drug repositioning; (**C**) Drug repositioning is the process of identifying new indication for known drug; (**D**) The approach of identifying new indication for known drug includes drug based, disease based or treatment based methods. The drug based approach consist of blinded or target based discovery of new indication while knowledge based discovery shared by drug based and disease based approach. Treatment based approach consist of target mechanism based discovery. Disease based and treatment based approach shares pathway based discovery [47].

Despite considerable progress in knockout technology the agents that target single-hit do not result in predicted behavior. This might be because the cellular system was more robust and single target having “back-up” system that responds in the absence of given target. Thus, the drug may not show desired response despite change in the cellular constituent. Instead of single target inhibition, partial or complete inhibition of few targets may be an effective approach. This implies multi-target drugs and combinatorial therapies may be a good strategy to design drug molecule against disease. For example, memantine was a multi-target drug (Figure 3A) used to treat Alzheimer’s disease [44]. Memantine was the first and only NMDARs (*N*-Methyl-d-Aspartate Receptor) antagonist approved by FDA. The success of memantine lies in its uncompetitive antagonistic mode of action where it blocks excessive NMDAR activity (IC50 = 1 μM) thereby, preventing the side effects due to complete inhibition of NMDARs. Similarly, recently development of SQ109 drug compound has shown to target multiple molecules act against Mycobacterium tuberculosis [45]. The success of this method was believed to be “weak linkage” of transcriptional, signaling and protein network. A “weak-linkage” describes the low affinity binding of target molecule partners with each other. As a result inhibition of number of molecules with low affinity not only influences the network but also affects linked biological pathways (Figure 3B). Sometimes the output is sufficient to achieve a significant modification which otherwise not possible with high-affinity target inhibiting drug compound [46]. Thus, multi-targets drugs and network approach might be an efficient way for drug development against complex diseases.

### 3.2. Repositioning

The term repositioning describes the new therapeutic utility of known drug compound (Figure 3C) [47]. The compound may show novel mode of action against a different disease in addition to the original intended disease. This suggests number of compounds that failed during clinical trials may show efficacy against one or more other diseases. Recently, concept of systematic repositioning has emerged as an application for drug development by performing analysis with multiple in vivo disease models. The idea is to screen drug candidate against several disease models. Several pharma companies use repositioning also known as therapeutic switching as an alternative approach to drug discovery with reduced risk of failure and lower cost. Figure S1 shows number of drugs repositioned. The drug repositioning of FDA approved drugs have shown efficacy against unmet medical problems. It accounts for about 30% of FDA approved drugs and vaccines [46]. Figure S1 indicates that the drugs have shown to act against wide range of diseases including nervous system disorders, cardiovascular diseases, metabolic disorders, and cancer. One of the well-known example sildenafil citrate (Viagra) used to treat hypertension was repositioned to treat erectile dysfunction [48]. Another drug molecule Xalkori (crizotinib) approved by FDA in 2011 was originally targeted to treat adult lung cancer. In addition to its original use it was found to be promising agent to treat two rare childhood cancers (lymphoma and neuroblastoma) [49]. Thus, repositioned drugs have capability to deliver alternative mechanism for many diseases. It has several advantages such as lower cost, reduced risk and shorter time as it can bypass different phases of drug discovery [50].

In 2012, NIH launched National Center for Advancing Translational Sciences (NCATS), which focused on drug repositioning through NCATS Pharmaceutical Collection database [51]. Recently, NCATS and pharma companies have agreed to use this database to explore new use of ~3800 known drug compounds by phenotypic drug discovery (www.ncats.nih.gov). Various techniques were used by these pharma companies to perform repositioning which includes blinded, knowledge based, or targeted-mechanism based approaches as shown in Figure 3D [47]. These methods can be customized according to available information and inventive steps. The pipeline starts with disease or a drug. For rare diseases where information is limited, phenotypic screening methods clubbed with drug libraries can be useful to validate in vivo efficacy for example drug astemizole against malaria [52]. In some diseases, prior knowledge available such as protein biomarker, priortorization of target-based or knowledge-based methods becomes useful. Here, knowledge based methods were used along with network based pathway analysis to reconstruct signaling networks. Subnetworks in these signaling networks were targeted with drug libraries and validated in vivo to come up with possible lead as validated for sunitinib drug molecule against brain metastasis [53]. If there exist more information about disease then either knowledge-based or signature-based methods along with omics analysis was deployed for drug-repositioning process as seen in case of lung adenocarcinoma targeting cimetidine drug [54]. In some studies different drug-dose combination have been used to identify treatment response. In these cases treatment omics data used to identify unknown targeted mechanisms or signaling pathways [47,55]. In summary, pharma industries can apply suitable approach for drug repositioning based on available information and screening techniques. The Biotechnology industry may take advantage of this approach to come up with desired outcomes thereby, optimizing the probability of drug compound against disease.

### 3.3. Molecular Imaging

In 1959 Russell and Burch have introduced 3R technique—replacement, reduction and refinement as guideline for use of animals in research [56]. The use of preclinical imaging follows the 3R technique. Molecular imaging was used to image molecular events at cellular and subcellular level in living systems. The changes at molecular level can be visualized and characterized in intact living organism. The imaging can be performed using molecular probes, which localize in specific tissue and allow exploring cellular changes in vivo. Recently it was used to detect and characterize disease, and evaluates treatment [57]. The advantages of molecular imaging against traditional drug discovery approach lies in (i) its repetitiveness (ii) non-invasive detection in intact living organism (iii) high success rate of identifying perturbation (iv) uniformity (v) reduction in animal cost and (vi) offering automatic analysis.

The preclinical imaging methods used on animals before moving to humans is positron-emission tomography (PET), single-photon-emission tomography (SPECT), magnetic resonance imaging (MRI) and optical imaging (OI) (Table 1) [58,59]. These techniques were capable to demonstrate the drug effect in model organisms by careful observation of molecular probe in the body. The observations studied in the model organism or genetically modified laboratory mice can be translated to humans. Thus, the technology aided to develop for personalized medicines. Clinicians can explore the drug effect in diseased model more effectively and quickly. Further, the techniques were invasive and the sensitivity of molecular imaging was at pm (pico-molar) level, ideal to focus drug effect on specific tissue. The imaging field is still evolving thereby, providing both opportunities and limitations, and can lead to new application, which seems infeasible today.

### 3.4. Translational Research

According to Mankoff et al. (2004) experimental models often do not translate to human pathology [60]. It was suggested that drug development is a two way process where ideas of scientific and clinical discipline are shared to achieve breakthrough in therapeutics, and that is translational research. The translational research well defined by APS (American Physiological Society) as “the transfer of knowledge gained from basic research to new and improved methods of preventing, diagnosing, or treating disease, as well as the transfer of clinical insights into hypotheses that can be tested and validated in the basic research laboratory” [61]. This definition describes that translational research is bidirectional, bench to bedside and bedside to bench to bring real therapies for patients. Translational research has two key components: integrative physiology and clinical physiology, which shares information among each other to bring the basic research to treat the diseases.

The “bench to bedside” concept describes research on identification of a disease biomarker and expression of which can be modulated by drug molecule in animal experiments [62]. The efficacy and safety is checked during pharmacodynamics and pharmacokinetics. The major advantage of the technique was that the research that lies dormant for unknown reasons could be translated to deliver its effect. By this way the appropriateness of the target can be enhanced, number of cycles can be reduced, and further help to define dosage levels in subsequent trials. Thus, translational research has the potential to overcome issues of drug discovery. However, the real answer is, validation of disease model [63]. To evaluate the efficacy of drug target one need to perform in vivo evaluation. The understanding of disease model itself can give detail insight of the cellular system and can be further correlated with human disease condition to prevent failure of drug in clinical trials. Bowes et al. (2012) have discussed role of in vitro pharmacological profiling in early identification of off-target pharmacological interactions causing market withdrawal of drug candidates and how the four major pharmaceutical companies such as AstraZeneca, GlaxoSmithKline, Novartis and Pfizer sharing the knowledge of minimal panel of targets to reduce safety-related attrition of drug molecules during drug discovery and development [64]. Similarly, Translational Medicine Guide (TxM Guide) was implemented by Merck (began in 2011) to facilitate lead optimization into its stage-gate drug development process. The major objective of TxM Guide to encourage strategic drug development thinking for each decision point from lead optimization to Phase II development [65]. AstraZeneca seems to establish a framework based on specific determinants like the right target, the right patient, the right tissue, the right safety and the right commercial potential [66]. However, it is too early to discuss which approach is better in long term.

### 3.5. Human Model Organism

Traditionally, mouse or rat remains to be the first choice of model organism for studying human diseases and to test the drug effect during the drug discovery process [31,67]. However, it was observed that these organisms can give crude extrapolations of toxicity and pharmacokinetics in humans, thereby making their use limited in interpreting the effect of a drug on a disease [68]. It was reported that more than 100,000 people die every year from adverse reactions caused by animal-tested drugs. Around 92% of new drugs fail in clinical trials though succeeded in animal tests. The report released by FDA suggests that nine out of ten experimental drugs do not reach final stage of validation (www.fda.gov). Few reports question use of animals for human diseases [68,69,70]. Wall and Shani (2008) have concluded in their report that “On average, the extrapolated results from studies using tens of millions of animals fail to accurately predict human responses” [71]. While a few justify that use of animals as prognostic models [72,73,74,75]. In the 1960s Thalidomide was introduced in market, which was a sedative, prescribed to pregnant women to help reduce morning sickness. However, use of Thalidomide lead to birth of children without limbs [76,77,78].

**Figure 4:**
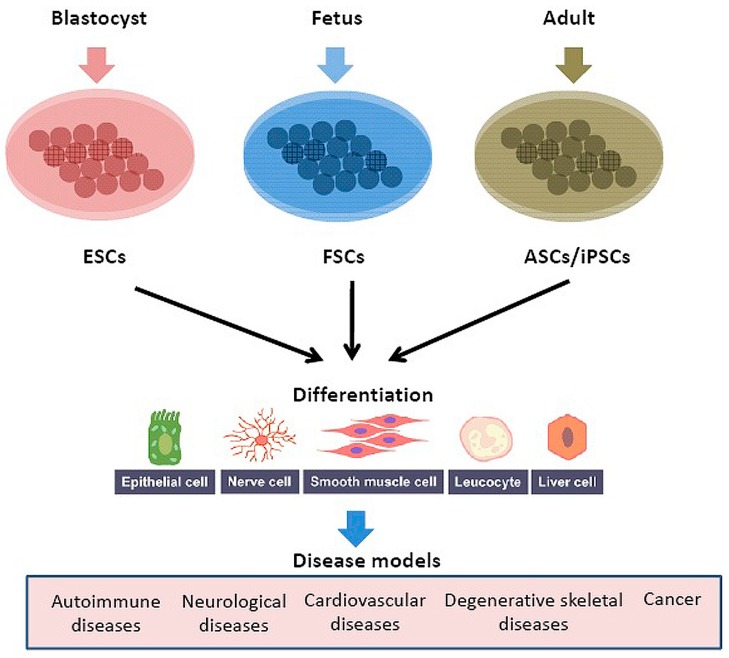
Use of human cells for stem cell-based assays and therapies. The embryonic stem cells (ESCs), fetal stem/progenitor cells (FSCs), adult stem/progenitor cells (ASCs) and induced pluripotent stem cells (iPSCs) used in regenerative medicines. These stems cells cultured and reprogrammed to desired cell type. After differentiation obtained desired cell types studied and tested for treating different diseases including autoimmune diseases, neurological diseases, cardiovascular diseases, degenerative skeletal diseases and cancer.

Focusing on human biology can address adverse reactions in humans, which were unnoticed in animal models [79]. Improved preclinical models can empower to access human biology. This includes the development of human tissue with the relevant disease in vitro by reprogramming cells can be termed as “body on a chip” (or “patient in a dish”). Thus, by mimicking the human organs the virtual organs can be created and studied for effect of drug (Figure 4). In 2011, Safety Evaluation Ultimately Replacing Animal Testing (SEURAT) program was initiated in Europe to develop alternative safety tests for cosmetics and chemicals (http://www.seurat-1.eu). The idea was to use stem cell-based assays for toxicity evaluation in heart, liver, muscle, skin, and nerves. Recently new methods and tools have been used to culture human cells, reprogram them to specific phenotype and perform clinical trials [79,80,81]. The study published by Cell Reports show that induced pluripotent stem cells (iPSCs) derived from patients with frontotemporal dementia were genetically corrected and converted to cortical neurons [82]. This shows regenerative medicines are new tools to model diseases and test drug molecules in induced pluripotent stem cells [81]. This approach was based on the idea of personalized medicines where patients own cells will be induced to specific lineage and used to test effect of drug molecule. In 1998, Trastuzumab (Herceptin) was the first genetically guided therapy introduced by FDA to treat breast cancer only if cancer patient tested positive for overexpression of the HER2/neu receptor [83]. In 2011, drug vemurafenib (Zelboraf) approved by FDA for treating metastatic melanoma patients with BRAF V600E mutation test [84]. In 2013, the FDA approved Tafinlar and Mekinist with a genetic test called the THxID BRAF test to diagnose patient with BRAF V600E or V600K mutation (www.fda.gov). In 2014, of 41 FDA approved NMEs, 9 NMEs (20%) were personalized medicines as classified by the Personalized Medicine Coalition (www.personalizedmedicinecoalition.org) (Table 2). This shows that the FDA is focusing on human response as paramount importance for the future of personalized medicines.

## 4. Lack of Innovation or Need of an Alternative Approach

Since last few decades, the attrition rate of the pharma industry has been considerably debated. Many analysis reports give the reason of attrition as a “lack of innovativeness” by the pharma sector [85,86]. In the 1990s, attrition was at ~40% and linked to poor bioavailability and pharmacokinetics [5]. In 2000, the reason of attrition was linked to the efficacy of drug molecule. If we look at the number of NMEs succeeded between 1996 and 2004, the trend seems decreasing. Inadequate pharmacology and the lack of predictability of animal models are said to be associated with attrition. Between 2004 and 2014, the NMEs approved by FDA increased but rate of attrition was still high in Phase 2. Investigation of pharma industry’s innovation record suggests though fluctuations in the approval of new NMEs the perception of “lack of innovativeness” was wrong. The innovation here does not only mean the successful launch of new drug but the identification of novel mechanism for treatment of disease. The innovation is inextricably interwoven from academic/industry findings, stage of development and funding to the eventual NME [87]. The key determinants of pharma R&D cost are success rate and development time [65,88]. It is important to know that the concept of innovation has changed from previous decades. The industry is now focusing on productivity along with safety, efficacy and novelty. Currently, pharma industries like AstraZeneca, GlaxoSmithKline, Novartis and Pfizer sharing the knowledge obtained from in vitro pharmacological profiling. The approach not only reduces the off-target pharmacological interactions but failure of high number of drug candidates during drug development [88]. Merck has implemented question-based Translational Medicine Guide (TxM Guide) to foster lead optimization process [65]. While, Chorus working as independent clinical development organization within Eli Lilly and Company focused on drug candidate selection [89]. Some of the industries are following “quick-kill” or “fast-fail” strategy to reduce late pre-clinical stage attrition [90].

**Figure 5:**
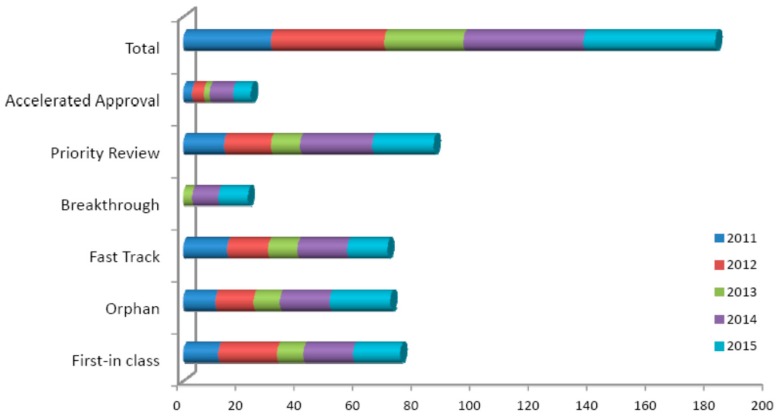
Innovative NMEs approved in 2011–2015. CDER used a number of regulatory methods including Fast Track, Breakthrough, Priority Review, and Accelerated Approval to expedite innovative NMEs to market.

In 2015, FDA approved 45 drugs (41 in 2014) whereas The European Medicines Agency (EMA), 39 drugs were approved compared to 40 in 2014 and 34 in 2013 (http://www.ema.europa.eu/ema/) [91,92]. The innovative drugs approved by EMA includes in Cancer (Blincyto, Farydak, Imlygic, Opdivo, Nivolumab BMS and Keytruda), Cardiovascular (Entresto, Repatha and Praluent), Haematology (Praxbind), Neurology (Wakix). Among 45 FDA approved novel drugs, 13 (9 in 2014) were based on concept of personalized medicines (www.fda.gov) [93]. More than half of approved drugs were Priority Review based (improvements over existing therapies). In addition, the “orphan drugs” approved for rare diseases were more than any of the previous years (21 out of 45). Further, 14 drugs (31%, 22% in 2014) approved by FDA were for cancer treatment. Among 45 NMEs, 14 (~31%) were First-in-Class (innovative nature of a drug) having novel mode-of-action for treating medical conditions over conventional therapies. This data shows 2011–2015 year was an indicator of innovative drugs than previous or any of other years (Figure 5).

## 5. Conclusions

Recent analysis of FDA approved first-in-class small-molecule drugs between 1999 and 2008 suggested that phenotypic screening strategies have been more productive than target-based approaches [11,93]. Of the 259 NMEs (1999–2008), 75 were found to be First-in-class molecules with novel molecular mechanism of action. Among them, 28 (37%) were identified from phenotypic screening and 17 (23%) were from the target-based approach. Based on this study question has been raised whether the shift to target-based drug discovery could be responsible for the decline in the productivity of the pharmaceuticals industry. Whereas the study carried out by Eder et al. shows that the out of 113 first-in-class molecules (1999–2013), 33 (30%) resulted from system-based approach and 78 (70%) were from target-based approach [10]. System-based approach included chemocentric (25, 23%) and phenotypic-screening (8, 7%) approach. Meanwhile, the target-based approach consists of small-molecule (45, 41%) and biologics (33, 30%). This study shows a significant increase in target-based approach identified first in class drugs. Though the results differ from both studies, the time frame for system-based and target-based NMEs was found to be around 25 and 20 years respectively. Advancement in genomics was thought to impact the target-based approach. However, looking at the NMEs approved in the year it seems industry needs to focus on system-based approach. The target-based approach could be a dominating factor, but over the period many factors have changed. We cannot discard the target-based approach but at the same time cannot rely only on a single target ignoring the complexity of cell. Although optimal drug discovery is difficult to achieve but synergy of different approaches such as multiple-target drug design, drug repositioning, molecular imaging, translational approach and regenerative medicines may provide better disease treatment. Multiple target drug approach could surpass complex method of single target identification. The future drug-design approach shall take into account next generation protein drugs with multiple fit and low affinity molecules. Thus, instead of relying on a single gene target the pharmaceutical companies should focus on drug development that can address multiple targets. Further, to increase flexibility and specificity the drug delivery systems adopting this multi-target approach would be beneficial. Repositioning of failed drug can provide alternative mechanism to combat different diseases and improve the portfolio value by ~10%. Translational approach can accelerate the identification of lead compounds and facilitate ‘bench to bedside’ method to fulfill medical need.

## Figures and Tables

**Table 1:** Various techniques are available for molecular imaging in disease and drug development.

Imaging	Sensitivity	Probe	Applications
Positron-emission tomography (PET)	pm	Positron emitting radiotracer	Cancers, heart disease, gastrointestinal, endocrine, neurological disorders, any abnormalities within the body
15O, 11C, 18F, 76Br			
Single-photon-emission tomography (SPECT)	pm	Gamma rays emitting radiotracer	Cancers, dopamine transporters (DAT), neurological diseases, pharmacokinetics and pharmacodynamics
123I, 99mTc			
Magnetic resonance imaging (MRI)	µm	Para- or superpara-magnetic radiotracer	Cancers, bleeding, injury, blood vessel diseases, or infection, cellular metabolism
Optical imaging (OI)	pm	Fluorophores	Gene expression

**Table 2:** FDA approved personalized medicines in 2014.

Drug	Disease	Influential Biomarker
Lynparza (olaparib)	Ovarian cancer	BRCA
Vimizim (elosulfase alpha)	Mucopolysaccharidosis Type IV	Type A or B
Cyrazma (ramucirumab)	Gastric or gastro-esophageal junction adenocarcinoma or non-small cell lung cancer (NSCLC)	EGFR or ALK
Zykadia (ceritinib)	Gastric or gastro-esophageal junction adenocarcinoma or non-small cell lung cancer (NSCLC)	ALK
Beleodaq (belinostat)	Peripheral T-cell lymphoma	UGT1A1
Cerdelga (eliglustat)	Gaucher disease type 1	CYP2D6
Harvoni (ledipasvir and sofosbuvir)	Chronic hepatitis C infection	Genotype 1
Viekira Pak (ombitasvir, paritaprevir, and ritonavir; dasabuvir)	Chronic hepatitis C infection	Genotype 1
Blincyto (blinatumomab)	B-cell precursor acute lymphoblastic leukemia (ALL)	Philadelphia chromosome biomarker

## Supplementary Materials

Supplementary materials can be found at http://www.mdpi.com/2227-9059/4/4/27/s1.
