# Supplementary figures and images for: Complementary Approaches to Existing Target Based Drug Discovery for Identifying Novel Drug Targets
Source: Biomedicines. 2016 Nov 21;4(4):27. doi: 10.3390/biomedicines4040027 (PMC5344266; doi:10.3390/biomedicines4040027)

## Suh​as Vasaikar, Pooja Bhatia, Partap G. Bhatia and Koon Chu Yaiw

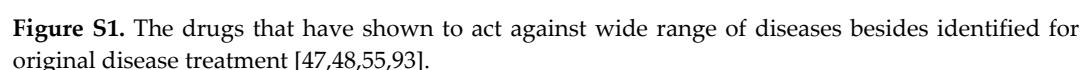

Supplement: Supplementary file 1 [file biomedicines-04-00027-s001.pdf]
